# Supplementary figures and images for: Comparative analysis reveals distinctive epigenetic features of the human cerebellum
Source: PLoS Genet. 2021 May 6;17(5):e1009506. doi: 10.1371/journal.pgen.1009506 (PMC8101944; doi:10.1371/journal.pgen.1009506)

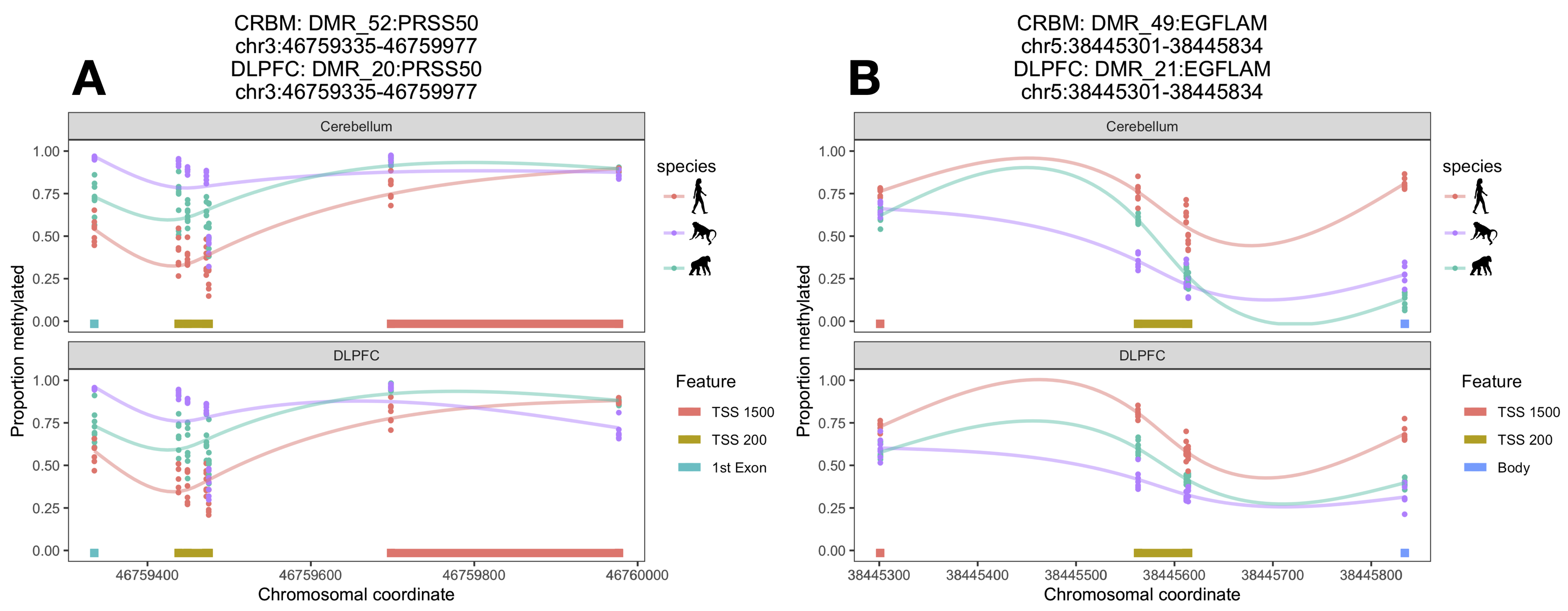

Supplement: S1 Fig — A = PRSS50 and B = EGFLAM. Lines are loess smoothed methylation values for each species across the DMR and each point represents raw methylation values for each individual at each CpG site within the DMR ranges. TSS200 = within 200 bp of a transcription start site and TSS1500 within 1500 bp of a transcription start site. Chromosomal coordinates are in base pairs and refer to human genome build hg19. (TIF) [file pgen.1009506.s001.tif]

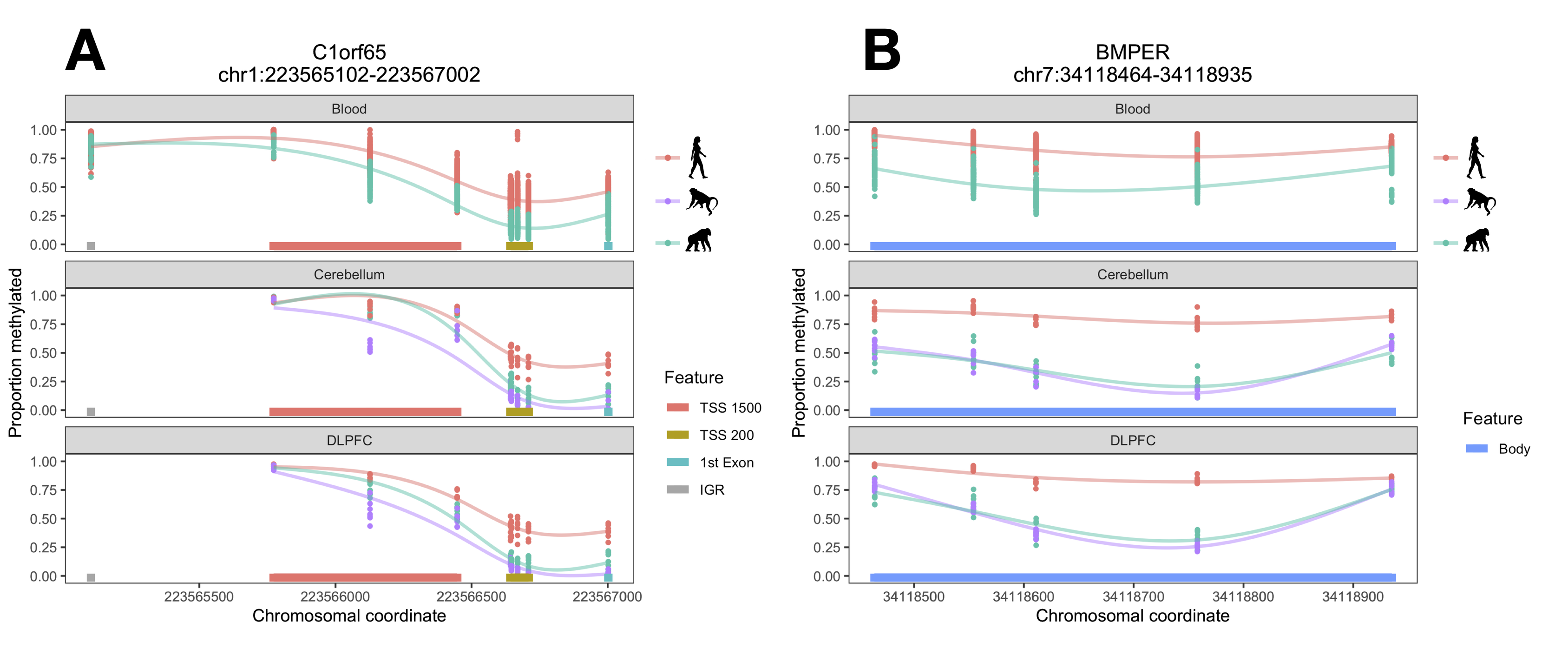

Supplement: S2 Fig — A = C1orf65 and B = BMPER. Lines are loess smoothed methylation values for each species across the DMR and each point represents raw methylation values for each individual at each CpG site within the DMR ranges. TSS200 = within 200 bp of a transcription start site and TSS1500 within 1500 bp of a transcription start site. Chromosomal coordinates are in base pairs and refer to human genome build hg19. (TIF) [file pgen.1009506.s002.tif]

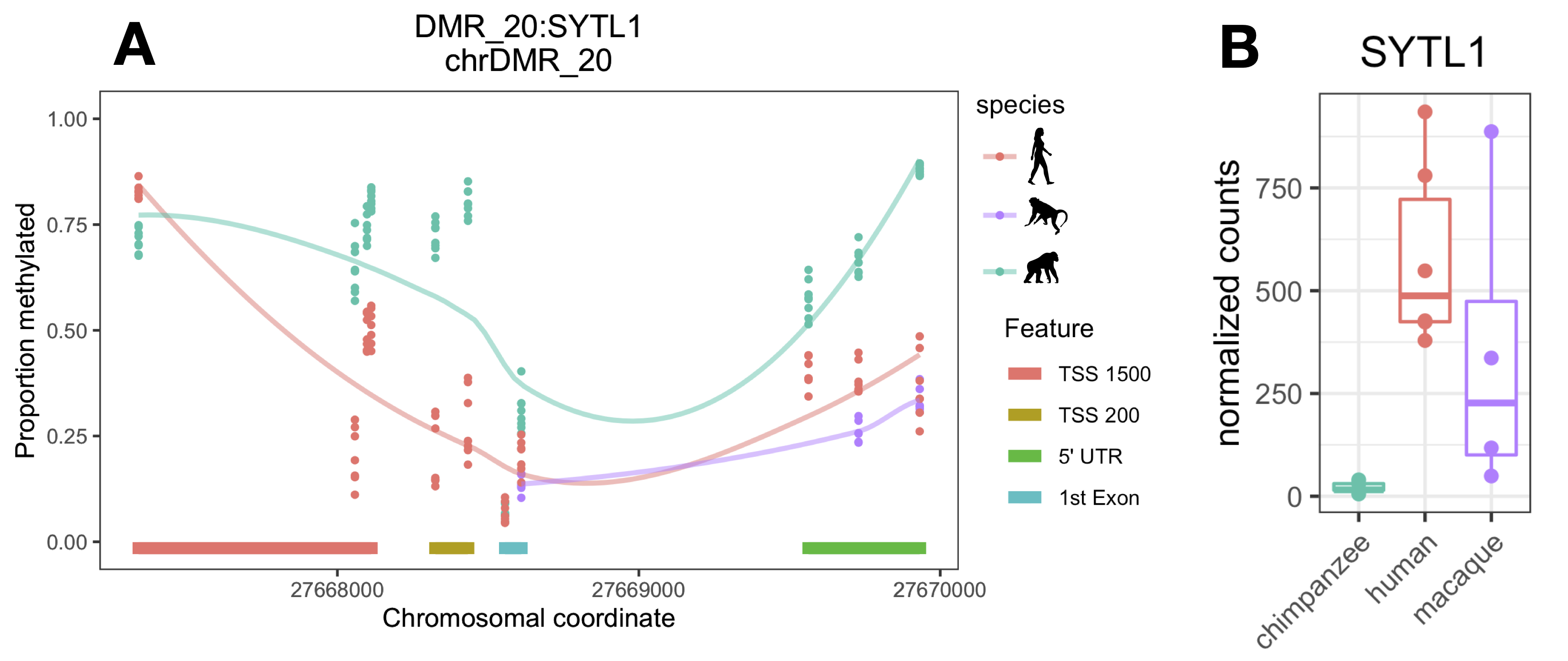

Supplement: S3 Fig — A = methylation, B = gene expression. Lines are loess smoothed methylation values for each species across the DMR and each point represents raw methylation values for each individual at each CpG site within the DMR ranges. TSS200 = within 200 bp of a transcription start site and TSS1500 within 1500 bp of a transcription start site. Chromosomal coordinates are in base pairs and refer to human genome build hg19. (TIF) [file pgen.1009506.s003.tif]

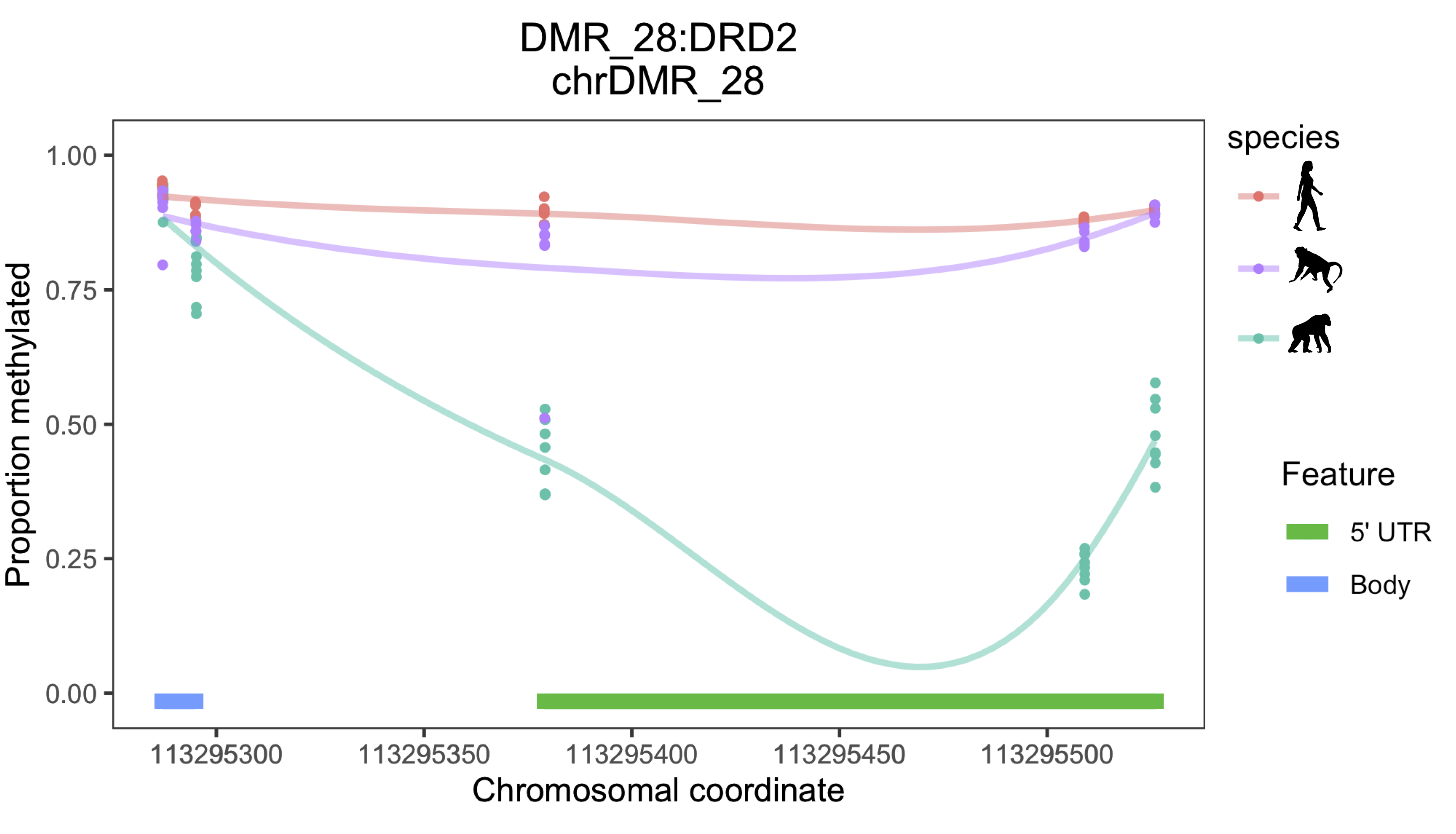

Supplement: S4 Fig — Lines are loess smoothed methylation values for each species across the DMR and each point represents raw methylation values for each individual at each CpG site within the DMR ranges. Chromosomal coordinates are in base pairs and refer to human genome build hg19. (TIF) [file pgen.1009506.s004.tif]
