# Supplementary material for: Comparative analysis reveals distinctive epigenetic features of the human cerebellum
Source: PLoS Genet. 2021 May 6;17(5):e1009506. doi: 10.1371/journal.pgen.1009506 (PMC8101944; doi:10.1371/journal.pgen.1009506)
Supplement: Attachment — Submitted filename: PGENETICS-D-20-01121_review.pdf [file pgen.1009506.s009.pdf]

Summary

The aim of this paper is to examine epigenetics related to the evolution of human neural specialization. For this research, the authors measure genome-wide DNA methylation levels in brain tissues from humans, chimpanzees, and macaques. Specifically, they sample from two regions of the brain (dorsolateral prefrontal cortex and cerebellum), and by comparing across species, they identify changes in methylation that are specific to the human lineage and changes in methylation that are specific to the chimpanzee lineage. They identify more human-specific methylation changes than chimpanzee-specific methylation changes in both tissues, and they find that some genes with human-specific methylation changes are involved in neurobiology functions. Further, they compare these findings to the results of several other previously published differential methylation and differential gene expression studies.

This work is likely of interest to researchers in genetics and genomics. However, several important aspects of the methodology are missing. In particular, the statistical analyses employed need to be more detailed, and potential confounding batch effects should be further considered. Additionally, as the authors note, research on DNA methylation in brain tissues from primates has been previously examined, so the originality of this study should be more explicitly stated and reinforced.

Below are areas that should be improved before publication.

Major Issues

Methods

- Study subjects
  - [lines 349-351] Does this mean that no samples had neurodegenerative diseases or brain trauma? Also, did any of the humans included in this study have any other neurological disorders (e.g., autism or schizophrenia)? This could affect the interpretation of some of the results (see lines 239-245).
  - [Figure 1A] Is it possible that cross-species sex differences impacted the methylation results (specifically that more human-specific methylation changes were identified)? Human samples include 1 female and 6 males, while chimpanzee and macaque samples are closer to 50/50.
- Tissue dissection
  - [lines 363-372] Is it possible that cross-species differences in preparation impacted the methylation results (specifically that more human-specific methylation changes were identified)? Human sample collections were done at a different facility and by different researchers than the chimpanzee and macaque sample collections. Also, RNALater was only used on chimpanzee and macaque samples, not human samples.
  - [lines 370-372] Is it possible that cross-species differences in tissue sampling locations impacted the methylation results (specifically that fewer chimpanzee-specific methylation changes were identified)? For chimpanzee sample collections, tissues came from both the right and left hemispheres, while human and macaque tissues came from just the left hemisphere.
- DNA extraction and microarray analysis
  - Were any replicate samples included on the EPIC array to validate the reproducibility of these data?
- Differential methylation analysis
  - What variables were included in the linear models (e.g., age, sex, species, tissue, batch)?
  - Were data analyzed separately for each tissue? If all data were analyzed together, did the model design account for multiple samples coming from the same individual?
  - How was potential cell heterogeneity accounted for (e.g., latent variables)?
  - What was the rational for picking a change in beta value of 0.15 threshold?

- Have alternative definitions of DMRs been considered? It seems odd to call DMRs if only 1 significant DMP is present in a region of CpGs. Do the number of DMRs identified decrease drastically when 2 significant DMPs are required?

- Brain structure specificity
    - It would be worthwhile to reinforce this comparison with a genome-wide assessment. Specifically, assess how well methylation levels in brain samples correlate with methylation levels in blood samples. The correlation between methylation levels of significant DMRs that overlap in brain and blood should be higher than the correlation between significant DMRs that do not overlap across tissues, which itself should be higher than the correlation between methylation levels of non-significant DMRs in each tissue (or the methylation levels across all 148,547 sites examined).

- Correspondence with gene expression
    - It would be worthwhile to reinforce this comparison with a genome-wide assessment. Specifically, assess how many differentially methylated genes also show differential expression and whether the changes in methylation/expression directions matches expectations (↑methylation in TSS = ↓expression / ↑methylation in gene body = ↑expression). This would help to isolate which changes in methylation might have direct, functional effects on gene expression.
    For example, instead of stating, "Many human-specific, hypomethylated DMRs (S1 and S2 Tables), which are likely to be associated with increased gene expression…" (lines 142-144), the analysis described above would reveal how many of these DMRs actually correspond with increased gene expression (and vice versa).

- Correspondence with previous studies
    - It would be worthwhile to reinforce this comparison with a genome-wide assessment (see comments regarding brain structure specificity methods above). Is it possible to access the data from these papers to do such a comparison?

Introduction

- Be consistent regarding the primary goal of this research throughout the manuscript.
The abstract implies that the goal is to investigate epigenetics related to the evolution of human neural specialization. However, neural specialization is not mentioned again in the paper, and the epigenetics of neural specialization is not explicitly examined. Rather, this study characterizes methylation in multiple regions of the brain from multiple species and evaluates how the variation identified relates to what is known about human evolution and neurobiology. If this is the central aim, then reinforce this idea throughout the entire manuscript. If this is not the central aim, then the manuscript needs to be reframed to make this clearer.
(Other thoughts on this topic: The brain regions being examined clearly have specialized roles in humans, but since these regions are also present in other primates, do they not also have similar functions in other primates? Further, since there is not a deep assessment of methylation changes that are unique to each brain tissue region, the specializations of these regions in comparison to one another does not seem to be the focus.)

- Be clear about what aspects of the study design and experimental questions make this research original and further advance the field.
Compared with Mendizabal et al. (2016), this paper does incorporate an extra region of the brain, as well as a slightly larger sample set. However, it also has lower data resolution (array-based method) than Mendizabal et al. (2016) (whole-genome bisulfite). This is not necessarily a concern, but without bolstering the originality of the research early on and throughout the manuscript, it becomes lost.

Results

- Differential methylation analysis
    - How many genes are associated with the DMPs/DMRs identified, and what genomic regions are DMPs/DMRs located in (e.g., TSS, exon, intergenic)?
    - [Table 2] Only 16 genes related to aspects of human-specific neurobiology are listed, which does not seem substantial. Are other identified genes not related to neurobiology? It would be worthwhile to

perform GO/KEGG functional enrichment analyses to reinforce the statement that human-specific methylation changes are predominantly involved in neurobiology functions.

- Brain structure specificity
    - Is it possible that the difference in overlap between DLPFC and the cerebellum (1/3 of human-specific DMRs vs. 1/5 of chimpanzee-specific DMRs, lines 156-158) is due to differences in cellular heterogeneity or other confounding batch effects?
- Correspondence with gene expression
    - In the enrichment tests, how are the null hypotheses defined? If the methylation changes are expected to have a functional effect on gene expression, shouldn't we expect most genes with differential methylation to also be differentially expressed?
    - [lines 170-171] It is stated that the human-specific DLPFC genes with shared differential methylation and differential expression show anticorrelated methylation and expression levels (what we expect). Are the DMRs associated with these genes in the TSS? Is this relationship also present for the cerebellum human-specific genes, as well as the chimpanzee-specific genes?

Discussion

- It is still unclear whether any methylation differences identified in this study are due to sex, age, collection conditions, processing effects, etc. that were different for humans as compared to chimpanzees and macaques. Either several of the claims in this section should be qualified, or evidence indicating that these factors are not contributing to the variation should be shown.
- [lines 316-320] Is the DRM identified in ESR1 human-specific? Does this change in methylation affect gene expression? Why do you think this gene is regulated differently across species?
- [lines 325-330] This qualification and recommendation for future work is well thought through.
- [lines 334-337] Isn't the modest correspondence between methylation and gene expression just indicating that several methylation changes identified do not have functional effects on gene expression?
- [lines 338-339] This phrase is ambiguous and should be rephrased. This study finds that more changes in methylation are identified along the human lineage than along the chimpanzee lineage. There is no comparison in the number of methylation changes identified in brain tissue as compared to other tissues.
- Is it possible that the human-specific changes in methylation identified are a result of different environmental exposures during life? Would this mechanism change the evolutionary significance of these findings?

Minor Issues

Supplemental Figures S2 and S3

- Some human-specific methylation and expression do not look human-specific (e.g., S3U, S3X). Why is this?
- Some human-specific methylation and expression levels are opposite what is expected (e.g., S2E and S2F, S3W and S3X). Why is this?
