# Supplementary material for: Comparative analysis reveals distinctive epigenetic features of the human cerebellum
Source: PLoS Genet. 2021 May 6;17(5):e1009506. doi: 10.1371/journal.pgen.1009506 (PMC8101944; doi:10.1371/journal.pgen.1009506)
Supplement: Attachment — Submitted filename: PLoS_Genetics_response_to_reviewer_comments_2021.01.09.docx [file pgen.1009506.s010.docx]

Editor's comments:

In this paper, the authors studied epigenetics related to the evolution of human neural specialization, measuring genome-wide DNA methylation levels in brain tissues from humans, chimpanzees, and macaques. Their findings of evolutionary changes in species-specific methylation with its implication to neurobiological function are significant and interesting. All the three reviewers agreed with this point.

However, Reviewers 2 and 3 questioned the authors’ usage of the “EPIC microarray” rather than whole-genome bisulfite sequencing for a less biased data set. In particular, Reviewer 2 suggested rejection of this manuscript, pointing out that the choice to use EPIC array to investigate the brain epigenomic and the absence of any cell (nuclei) type enrichment significantly reduced the potential impact of this study. Reviewer 3 also stated that the major methodological issue that one see with this study is profiling methylation sites by microarray rather than whole-genome bisulfite sequencing for a less biased data set.

Therefore, I would like to see the authors’ response to the above-mentioned points regarding the methodology.

Moreover, I have to agree with the following particular points of Reviewers 1 and 3:

As Reviewer 1 pointed out that the statistical analyses employed need to be more detailed, and potential confounding batch effects should be further considered. This Reviewer further continued that additionally, as the authors note, research on DNA methylation in brain tissues from primates has been previously examined, so the originality of this study should be more explicitly stated and reinforced.

Reviewer 3 also stated that a more focused approach would have served this study better: Identify the areas of greatest difference between species, narrow down on the most interesting or understudied pathways, and experimentally test whether hypo- or hypermethylation correlates with increased or decreased expression of the genes in question to construct narrow hypotheses around these specific data.

We are grateful for the detailed feedback from the reviewers, as well as the editor’s synthesis of the major points requiring the most attention. We have addressed these concerns as described below in direct response to the reviewers’ specific comments. We further elaborate here that we agree with the reviewers that there are certain limitations to methylation microarray data, in particular the bias introduced by using a technology designed to target the human genome to assay methylation in non-human primate species. Further, there are many intergenic regions that our analysis will miss entirely. However, we believe that careful analysis of the resulting data by restricting analysis to sites bioinformatically predicted to hybridize efficiently in all three primates and an awareness of the array’s limitation can largely allow these limitations to be overcome. In our reanalysis of our data for this revision, we have implemented even more stringent filtering of probes (e.g., removing probes in which SNPs have been identified within 5 bp of the target CpG site in nonhuman primates). This of course will further restrict our analysis of conserved regions, which is a form of bias; it does not mean however that our results are not valid and, indeed, comparative evolutionary studies often inevitably suffer this sort of bias – toward conservation, toward regions in which we are confident of homology, etc. In our revised manuscript, we abandon any attempt to draw conclusions about the raw numbers of differentially methylated probes and regions amongst the different species, recognizing that due to the very partial picture our data produce, such comparisons are unlikely to be very meaningful. We further note that besides the limitations of the array, it has certain advantages, such as allowing for more direct comparison with previously generated data, given that arrays have been the predominant technology for human methylation profiling, and increased sensitivity, and that BS-seq methods usually result in limited depth of coverage and uniformity of coverage across sites and samples unless sequenced to extremely high depth, which remains prohibitively expensive [1]. We thus selected a cost-effective method and present data for a larger number of samples per species than for previous comparative primate brain epigenetic studies. While we acknowledge our study’s real limitations and look forward to conducting sequencing-based studies in the future, we are confident our data contribute informative results to the field and are likely to provide a useful basis for future studies (for example, targeted studies).

We further agree with the reviewers that assaying heterogeneous tissues presents further limitations. In our revised manuscript, we better address the question of cell-type heterogeneity in our brain samples by using a reference-free approach to account for cell composition variation given the lack of appropriate references (described on lines 442-454).

We have also tried better to highlight the originality of our study throughout (for example, lines 114-133). In particular, our study benefits from an increased sample size relative to most previous studies and, while these had focused on the neocortex, our work includes an important brain region, the cerebellum, that has been understudied and is gaining increasing recognition in comparative and evolutionary neuroscience for its likely importance in human traits, including manual dexterity and language [2]. These results are especially important in lending context to previous studies, as we identified two DMRs overlapping genes in the cerebellum that have been previously found to exhibit human-specific methylation in the prefrontal cortex.

Finally, we appreciate the suggestion of a more focused approach and we have increased focus on the novelty of the cerebellum analyses in the revised manuscript. We also focus more specifically on DMRs that do not show overlap among brain structures/tissues and on DMRs that overlap putative regulatory elements. In addition, in response to other comments, we have increased the stringency of our analyses (and also added key methodological detail that was missing) which have narrowed down our results, which has functioned to focus the manuscript on the results that are most likely to be robust and biologically meaningful.

**Reviewer #1**

**Summary**

The aim of this paper is to examine epigenetics related to the evolution of human neural specialization. For this research, the authors measure genome-wide DNA methylation levels in brain tissues from humans, chimpanzees, and macaques. Specifically, they sample from two regions of the brain (dorsolateral prefrontal cortex and cerebellum), and by comparing across species, they identify changes in methylation that are specific to the human lineage and changes in methylation that are specific to the chimpanzee lineage. They identify more human-specific methylation changes than chimpanzee-specific methylation changes in both tissues, and they find that some genes with human-specific methylation changes are involved in neurobiology functions. Further, they compare these findings to the results of several other previously published differential methylation and differential gene expression studies.

This work is likely of interest to researchers in genetics and genomics. However, several important aspects of the methodology are missing. In particular, the statistical analyses employed need to be more detailed, and potential confounding batch effects should be further considered. Additionally, as the authors note, research on DNA methylation in brain tissues from primates has been previously examined, so the originality of this study should be more explicitly stated and reinforced.

Below are areas that should be improved before publication.

We thank the reviewer for the very detailed and thoughtful review of our manuscript, which has brought up many important points that we are grateful to have been led to consider. Addressing and incorporating these points has greatly improved our study.

**Major Issues**

Methods
• Study subjects

o [lines 349-351] Does this mean that no samples had neurodegenerative diseases or brain trauma? Also, did any of the humans included in this study have any other neurological disorders (e.g., autism or schizophrenia)? This could affect the interpretation of some of the results (see lines 239-245).

That is correct. The human samples from the NIH NeuroBioBank were all listed as control. We have added this to the Methods (line 391).

o [Figure 1A] Is it possible that cross-species sex differences impacted the methylation results (specifically that more human-specific methylation changes were identified)? Human samples include 1 female and 6 males, while chimpanzee and macaque samples are closer to 50/50.

This is an excellent point and we now include sex in our model.

• Tissue dissection

o [lines 363-372] Is it possible that cross-species differences in preparation impacted the methylation results (specifically that more human-specific methylation changes were identified)? Human sample collections were done at a different facility and by different researchers than the chimpanzee and macaque sample collections. Also, RNALater was only used on chimpanzee and macaque samples, not human samples.

This is a great point. We do not think that these types of methodological artifacts are likely to play a major role in our results, given that, unlike ephemeral RNA, methylation marks are relatively stable and robust to storage condition [3–6], so much so that, for example, reliable age estimates from methylation age clocks can be obtained from forensic samples [7]. Nevertheless, it is of course entirely possible this could have added an additional source of variance in our data and we now mention this the discussion (lines 362-368).

o [lines 370-372] Is it possible that cross-species differences in tissue sampling locations impacted the methylation results (specifically that fewer chimpanzee-specific methylation changes were identified)? For chimpanzee sample collections, tissues came from both the right and left hemispheres, while human and macaque tissues came from just the left hemisphere.

This is an interesting possibility. Due to the reasons given above, we do not think that sampling location is a major factor. Additionally, studies of gene expression have not tended to find very pronounced hemispheric differences in adult primate brain samples [8,9]. However, we have included hemisphere in the model.

• DNA extraction and microarray analysis
o Were any replicate samples included on the EPIC array to validate the reproducibility of these data?

Unfortunately, we did not assay biological replicates in this study. These postmortem primate brain tissues are extremely scarce and, in particular, given the small size of the cortical region sampled, additional tissue to provide replicates is not available [10]. We do think that the general consistency within individuals of the same species (i.e., as shown in the clustering in Fig 1) provides some assurance.

• Differential methylation analysis

o What variables were included in the linear models (e.g., age, sex, species, tissue, batch)?

We included species, age, sex, and hemisphere (lines 472-474).

o Were data analyzed separately for each tissue? If all data were analyzed together, did the model design account for multiple samples coming from the same individual?

Data were analyzed separately for each tissue (lines 459-460, 469-472).

o How was potential cell heterogeneity accounted for (e.g., latent variables)?

We have tried to address potential cell type heterogeneity by using a reference-free (latent variable) approach using the R package sva (lines 442-454).

o What was the rational for picking a change in beta value of 0.15 threshold?

Most studies use between 0.1 and 0.2 [11–16].

o Have alternative definitions of DMRs been considered? It seems odd to call DMRs if only 1 significant DMP is present in a region of CpGs. Do the number of DMRs identified decrease drastically when 2 significant DMPs are required?

We agree this is not intuitive. We first identified significant and human-unique DMPs using limma: linear models were fit for each CpG and CpGs for which the human-chimpanzee and human-macaque comparisons were significant and the human-chimpanzee and human-macaque deltaBeta > 0.15 were retained. To identify DMRs, we used the R package DMRcate to call DMRs, which takes linear models fit for each CpG as input, but calls DMRs based on Gaussian kernel smoothed methylation levels across stretches of the genome [17]. Thus, we ended up with some DMRs that only had a single CpG that met our criteria for being considered a significant, human-specific DMP. In our revised analysis, we have increased our stringency in calling DMRs in an effort to focus on the results most likely to be reliable and biologically meaningful. Specifically, we have only retained DMRs containing at least three CpGs meeting our criteria of significance (Benjamini-Hochberg adjusted p-value < 0.05 for both the human-chimpanzee and human-macaque comparisons) and difference in methylation (deltaBeta > 0.15 between humans and the other two species).

This does reduce our number of DMRs. We feel the remaining DMRs are mostly like to be biologically meaningful and thus warranting future study, and, to that end, focus in our manuscript.

• Brain structure specificity

o It would be worthwhile to reinforce this comparison with a genome-wide assessment.
Specifically, assess how well methylation levels in brain samples correlate with methylation levels in blood samples. The correlation between methylation levels of significant DMRs that overlap in brain and blood should be higher than the correlation between significant DMRs that do not overlap across tissues, which itself should be higher than the correlation between methylation levels of non-significant DMRs in each tissue (or the methylation levels across all 148,547 sites examined).

This is a great point and we have estimated correlation coefficients for average global methylation between the two brain structures and each of the two brain structures and blood. Reassuringly, these results are similar to previous studies (lines 169-173) [18].

• Correspondence with gene expression

o It would be worthwhile to reinforce this comparison with a genome-wide assessment.
Specifically, assess how many differentially methylated genes also show differential expression and whether the changes in methylation/expression directions matches expectations (↑methylation in TSS = ↓expression / ↑methylation in gene body = ↑expression). This would help to isolate which changes in methylation might have direct, functional effects on gene expression.

*For example, instead of stating, “Many human-specific, hypomethylated DMRs (S1 and S2 Tables), which are likely to be associated with increased gene expression...” (lines 142-144), the analysis described above would reveal how many of these DMRs actually correspond with increased gene expression (and vice versa).*

We agree with the suggestion. We have focused our revised manuscript on DMRs that cover regulatory regions and discuss whether the direction of gene expression is expected (lines 174-178, 181-201, 512-516).

• Correspondence with previous studies

o It would be worthwhile to reinforce this comparison with a genome-wide assessment (see comments regarding brain structure specificity methods above). Is it possible to access the data from these papers to do such a comparison?

We appreciate this suggestion and attempted this additional analysis. We were able to access the Zeng et al. 2012 WGBS dataset for humans and chimpanzees and identified all CpGs that overlapped between this dataset and our dataset (Methods lines: 531-540) in the dorsolateral prefrontal cortex. We found a high correspondence in methylation (average methylation for each species was >0.9) (Results lines: 202-205 and Discussion lines: 274-277).

Introduction

- Be consistent regarding the primary goal of this research throughout the manuscript.
  The abstract implies that the goal is to investigate epigenetics related to the evolution of human neural specialization. However, neural specialization is not mentioned again in the paper, and the epigenetics of neural specialization is not explicitly examined. Rather, this study characterizes methylation in multiple regions of the brain from multiple species and evaluates how the variation identified relates to what is known about human evolution and neurobiology. If this is the central aim, then reinforce this idea throughout the entire manuscript. If this is not the central aim, then the manuscript needs to be reframed to make this clearer.

*(Other thoughts on this topic: The brain regions being examined clearly have specialized roles in humans, but since these regions are also present in other primates, do they not also have similar functions in other primates? Further, since there is not a deep assessment of methylation changes that are unique to each brain tissue region, the specializations of these regions in comparison to one another does not seem to be the focus.)*

We appreciate this suggestion and reframed our manuscript to communicate our aims and how they connect to larger questions more clearly. In particular, we focus now more on the cerebellum, which has not been the subject of much comparative molecular research and thus is more novel. We explain how the cerebellum may play a role in human specializations like language and cultural behavior (lines 114-126). We connect this with methylation, much of which is the result of species- and cell type-specific developmental transcriptional programs, which presumably give rise to species differences (lines 78-81). We then connect this with the genes we discover to show human-specific methylation in our results (lines 369-372).

We agree that homologous brain structures are likely to play similar roles in different primate species. However, the field of evolutionary neuroscience has shown that conserved structures have been modified for unique functions specific to humans given that humans do not show whole new brain structures compared to our closest relatives. We make this more explicit on lines 88-95.

- Be clear about what aspects of the study design and experimental questions make this research original and further advance the field.
  Compared with Mendizabal et al. (2016), this paper does incorporate an extra region of the brain, as well as a slightly larger sample set. However, it also has lower data resolution (array-based method) than Mendizabal et al. (2016) (whole-genome bisulfite). This is not necessarily a concern, but without bolstering the originality of the research early on and throughout the manuscript, it becomes lost.

We appreciate this important suggestion and have tried to better highlight the novelty of the new brain region in particular throughout the manuscript, particularly in the Introduction (lines 114-126) and end of the Discussion (lines 208-213, 369-385).

Results

- Differential methylation analysis

o How many genes are associated with the DMPs/DMRs identified, and what genomic regions are DMPs/DMRs located in (e.g., TSS, exon, intergenic)?

We have added in information on how many DMRs are associated with putative regulatory regions to the Results section and (lines 174-178) and Supplementary Table S2 of our revised manuscript contains a list of all the genes and the genomic elements associated with the set of DMRs.

o [Table 2] Only 16 genes related to aspects of human-specific neurobiology are listed, which does not seem substantial. Are other identified genes not related to neurobiology? It would be worthwhile to perform GO/KEGG functional enrichment analyses to reinforce the statement that human-specific methylation changes are predominantly involved in neurobiology functions.

We performed an enrichment analysis and found that no terms are enriched at a 0.05 FDR, likely due to the retention of few probes after increasing our filter stringency.

• Brain structure specificity

o Is it possible that the difference in overlap between DLPFC and the cerebellum (1/3 of human-specific DMRs vs. 1/5 of chimpanzee-specific DMRs, lines 156-158) is due to differences in cellular heterogeneity or other confounding batch effects?

This is an interesting point that we have given careful consideration. Although we account for variability among individuals within regions in cell type heterogeneity, there is not currently an adequate method available to account for differences among regions in a reference-free manner, which are known, and the degree of difference could very likely be species-specific. However, based on what we know about cell type composition variation among brain structures and species, we would expect less overlap among structures in humans. The glia/neuron ratio in the prefrontal cortex increases with increasing brain size, but the proportion of cell types in the cerebellum stays fairly similar such that the two regions should be more similar in proportions in chimpanzees than humans [19,20]. Another possibility is that the bias of the array toward human enhancers may bias toward regions that will show greater similarity across tissues in humans. We now discuss these possibilities in the discussion (lines 298-299).

• Correspondence with gene expression

o In the enrichment tests, how are the null hypotheses defined? If the methylation changes are expected to have a functional effect on gene expression, shouldn’t we expect most genes with differential methylation to also be differentially expressed?

This is an important question. The null hypothesis is that methylation does not affect gene expression. We have retained this null hypothesis as, based on prior studies [3,10,21], we do not necessarily expect methylation at most genes to have very much effect on gene expression at a given moment. This is discussed in the Discussion (lines 314-345).

o [lines 170-171] It is stated that the human-specific DLPFC genes with shared differential methylation and differential expression show anticorrelated methylation and expression levels (what we expect). Are the DMRs associated with these genes in the TSS? Is this relationship also present for the cerebellum human-specific genes, as well as the chimpanzee-specific genes?

This is important information for interpretation. We now focus on only DMRs that overlap putative regulatory elements. Of the few genes that show corresponding gene expression changes, some show the canonical inverse correlation between methylation and expression; however, others do not. We have added these details to the results (lines 181-201) and also discuss how the direction of effect of methylation on gene expression can often still not be predicted (lines 326-333).

Discussion

- It is still unclear whether any methylation differences identified in this study are due to sex, age, collection conditions, processing effects, etc. that were different for humans as compared to chimpanzees and macaques. Either several of the claims in this section should be qualified, or evidence indicating that these factors are not contributing to the variation should be shown.
- We have now added in details on covariates (lines 472-474), as well as caveats to our discussion (lines 346-368).
- [lines 316-320] Is the DRM identified in ESR1 human-specific? Does this change in methylation affect gene expression? Why do you think this gene is regulated differently across species?
- This gene is no longer differentially methylated in our more stringent analysis.
- [lines 325-330] This qualification and recommendation for future work is well thought through.
- We are glad the reviewer viewed this section favorably.
- [lines 334-337] Isn’t the modest correspondence between methylation and gene expression just indicating that several methylation changes identified do not have functional effects on gene expression?
- This is correct. We have elaborated on our discussion of the relationship between methylation and gene expression, include helpful context from previous, similar studies (lines 314-345).
- [lines 338-339] This phrase is ambiguous and should be rephrased. This study finds that more changes in methylation are identified along the human lineage than along the chimpanzee lineage. There is no comparison in the number of methylation changes identified in brain tissue as compared to other tissues.
- We have removed this statement as we no longer include an analysis of which lineages show the most change, in recognition of the partiality of our dataset and its bias toward likely human-specific functional regions.
- Is it possible that the human-specific changes in methylation identified are a result of different environmental exposures during life? Would this mechanism change the evolutionary significance of these findings?
- This is a great question that we do not currently believe is possible to address with this dataset. We have included this important point, however, in our discussion (lines 353-362).

**Minor Issues**

Supplemental Figures S2 and S3

- Some human-specific methylation and expression do not look human-specific (e.g., S3U, S3X). Why is this?
- We no longer include this figure in the revised manuscript as some of these genes are no longer in our “regDMR” set now that we use more stringent thresholds for identifying DMRs and focus solely on DMRs that cover putative regulatory regions.
- Some human-specific methylation and expression levels are opposite what is expected (e.g., S2E and S2F, S3W and S3X). Why is this?
- Although we no longer include this figure in our revised manuscripts for the reasons given above, sometimes expression levels can be opposite to what would be expected based on methylation. This could be due to several reasons: 1) methylation can sometimes actually lead to increased gene expression, 2) methylation may sometimes have no effect or no longer any effect on gene expression and gene expression levels are driven by other regulatory mechanisms, 3) methylation’s repressive effect may be counteracted or overwhelmed by other regulatory mechanisms under some circumstances, or 4) in hypomethylated regions corresponding to activated transcriptional states, the gene may simply not be being transcribed at that moment and/or very highly transcribed, given that transcription tends to be intermittent [22] and hypomethylation, considered “transcriptionally permissive” but not necessarily predictive of transcription [23,24], can be associated with either activity or inactivity.

**Reviewer #2**

The study by Guevara et al, compared DNA methylation pattern for two brain regions: DLPFC, cerebellum across human, chimpanzees, and an old-world monkey rhesus macaques. This was a well-designed study to investigate the evolution of the epigenome and gene regulation in primates. The study included an impressive set of primary brain tissue specimens, with sample quality control and dissection carefully performed. In addition, DLPFC and cerebellum samples were collected from paired individuals, increasing the consistency of the dataset, and potentially allow the controlling of individual sample difference with regression models. However, the choice to use EPIC array to investigate the brain epigenomic and the absence of any cell (nuclei) type enrichment significantly reduced the potential impact of this study.

We appreciate the reviewer’s favorable view of the study aims, as well as the reviewer’s critique of the use of the microarray for profiling methylation. We agree that this presents a variety of important limitations, which we attempt to address below and in the revised manuscript.

1. A number of groups (e.g. Guo 2013 Nat Neuro, Lister 2013 Science, Gabel 2015 Nature) have reported the presence of a large quantity of non-CG methylation in mammalian brains, which appears to be a very unique characteristic of the brain compared to other tissues. Likely non-CG methylation is relevant to the evolution of the brain given its uniquely high level of accumulation in the brain. Given these findings, the authors should consider using whole-genome bisulfite sequencing that can more effectively measure non-CG methylation. If cost is a concern here, what about EPIC capture sequencing assay?

We agree with the reviewer that non-CG methylation is an interesting part of the picture that we were not able to address. We are investigating the possibility of using the EPIC capture approach, but currently have a limited ability to perform additional lab work due to the Covid-19 pandemic. We have added the caveat that our study was limited to CpG methylation to the Discussion (lines 350-353).

2. It is well known that brain cell types have very diverse methylation profiles, especially between neuronal cells and glial cells. It is fully feasible to separate neuronal and non-neuronal cells using anti-NeuN antibody. Kozlenkov et al., 2015 NAR has also developed a method to separate excitatory and inhibitory neuronal nuclei using antibodies against transcriptional factor proteins. These methods have been tested with primary human brain tissue and should be readily applicable to primate brain tissues.

We agree that cell type-specific methylation profiles are of great interest and appreciate the reviewer highlighting these specific, helpful methods. We are planning to incorporate this method into our future work, but we currently have a limited ability to perform additional lab work due to the Covid-19 pandemic. In the meantime, we have attempted to address the confound of cell type heterogeneity (see response to subsequent comments below).

3. Separating neurons and glia would be important not just for extracting cell type specific signature, but also to ensure the inter-species difference was not due to the variable ratio between neuronal and non-neuronal cells. Differences in cell-type composition is a major confounding factor for epigenetic epidemiology studies and may have an impact on inter-species comparison too.

This is a crucial point that we agree with completely and have acknowledged this limitation in our discussion (lines 300-313). To address this issue better, we have applied a reference-free, surrogate variable-based approach to correct for variation in cell type heterogeneity (discussed on lines 442-451).

4. The manuscript should include some technical description of the specimens, the method for methylation profiling and a brief discussion of the statistical method for DMR calling at the beginning of the result section. It was difficult to put the scale of DMR numbers in the context with no technical description at all in the main text.

We have included a new supplementary table (Supplementary Table S1) that contains more information on the specimens.

We have provided more detail on the DMR calling, which we also adjusted to be more stringent after considering the reviewers’ comments (lines 457-489).

**Reviewer #3**

The manuscript by Guevara et al. presents a relevant contribution to species-and tissue-related differences in global methylation of the dorsolateral prefrontal cortex and lateral cerebellum, brain areas considered of specific importance in the evolution of human cognition. Identified differentially methylated regions included genes that encode factors relevant to energy metabolism, neuroinflammation, neural degeneration, and neurodevelopment. Diminishing the impact of these discoveries, human-specific differentially methylated regions showed extensive overlap with those identified in blood, suggesting that some epigenetic marks may be common to nonneural cell types. Further diminishing the impact of this work, only modest correspondence between human-specific differential methylation and differential gene expression was observed. While the overall scope of work appears appropriate and some new insight may have been gained here, overall advancement for the field appears modest and somewhat dampens enthusiasm for this paper. It appears to me that a more focused approach would have served this study better: Identify the areas of greatest difference between species, narrow down on the most interesting or understudied pathways, and experimentally test whether hypo- or hypermethylation correlates with increased or decreased expression of the genes in question to construct narrow hypotheses around these specific data. Please see below a more detailed critique.

We thank the reviewer for the constructive attention to our manuscript. We agree with the reviewer that the overlap in DMRs between brain and blood, as well as the lack of correspondence of methylation with gene expression limit our ability to interpret the functional significance of some of the human-specific differences we identified. However, we do believe these results are nevertheless valuable for interpreting other studies identifying human-specific differences in bulk methylation of cortex that have been published over the years (e.g., [25]). We have additionally re-analyzed our dataset using higher stringency filtering (for example, removing probes with known SNPs in the nonhuman primates and requiring DMRs to contain at least three CpGs individually showing a difference in methylation of at least 0.15). This has led to a reduction in overlap among tissues.

We would also note that the lack of correspondence with gene expression is consistent with previous studies and may still provide insights into developmental differences in gene expression [3,10,21,26].

We agree that for the field to advance further, our more promising results will need to be functionally validated. However, we currently have a limited ability to perform additional lab work due to the Covid-19 pandemic.

The major methodological issue that I see with this study is profiling methylation sites by microarray rather than whole-genome bisulfite sequencing for a less biased data set. The authors explain the problem themselves “Because the Illumina methylation microarrays are designed to target human sites, we limited all analyses to CpG sites that should also be efficiently targeted in chimpanzees and rhesus macaques.” The bias that this approach introduces cannot possibly overstated and may stood in the way of discovering overlooked aspects of human-specific methylation and gene regulation.

We agree that the EPIC array introduces bias and limits the scope of our results. However, we do still believe our findings are of value and interest. Indeed, many studies have used Illumina arrays to study species differences in methylation [21,27,28]. Studies of nonhuman primates and even more distantly related mammals have also validated and made use of the EPIC array [29,30], as it represents a cost-effective way to generate ample, high coverage data. Our results represent an advance relative to prior studies of species differences in the brain, as the EPIC array targets many more sites than arrays previously used (i.e., the 27k or 450k arrays). Moreover, targeting the same sites across the genome lends itself to straightforward interpretation and comparison with other human datasets, given that most human genome-wide methylation data has been generated using the Illumina microarrays.

In an attempt to eliminate spurious findings and/or misinterpretations of the results, we have removed from our report and discussion of species differences in the overall amount of lineage-specific methylation, recognizing that the partiality of the dataset and the bias toward human functional enhancers makes this comparison not particularly meaningful. We have further increased the stringency of both the probes we include as well as how we call DMRs.

We mention these limitations in the Discussion (lines 346-368).

The experimental design remains somewhat obscure and I recommend the authors provide additional clarifications. I assume that the numbers of DMPs and DMRs provided inn Fig. 1 and Table 1 are in relation to the reference methylome of macaque, but nowhere is this clearly explained. Please clarify.

We appreciate this point and have added increased detail to the Methods section (lines 442-489). We have removed the analysis of the raw numbers of DMPs including in Figure 1 for the reasons stated above.

The authors discuss multiple very interesting pathways involving genes that associate with DMRs, such as glucose metabolism, neuroinflammation etc., but do not provide data on whether the genes in question are upregulated or downregulated in these cases, which seems to me the most relevant question.

This is a great point. In the Results and Discussion, we now make clear which genes show changes in expression (lines 181-201), recognizing that correspondence between gene expression and methylation is often modest and that many of these genes do not show human-specific expression, as discussed in the Discussion (lines 314-333).

I recommend that whenever in the results the number of overlapping DMRs is provided, to also indicate what percentage of the total relevant DMRs this number corresponds to.

Another great point. We now include percentages in the Results (lines 165-168).

Results of figures 3 and 4 are only mentioned in the discussion and there only partially. Please present these data in the results section as well.

We now mention all figures in the text.

In the discussion the authors state: “Many human-specific DMRs also overlapped genes involved in neurodevelopment and neurodevelopmental disorders, especially autism spectrum disorder (ANKS1B, GCNT2, DIP2C, RAI1, MYOZ3, DIP2C, DLGAP1, ZNF8) and schizophrenia (NT5DC2, SNED1, RAI1, MYOZ3, FAM193A).” How do the listed genes associate with the ID and ASD modules established by Parikshak et al. 2013? Are prenatal or postnatal modules of greater relevance?

We thank the reviewer for pointing us to this interesting paper. It does not seem that any of our genes overlap with the genes among their primary results. However, some similar genes are reported (e.g., *DLGAP1* in our study and its family member, *DLGAP2*, in their study). They also identify the pathway of synaptic development in their study, which is consistent with our results.
