# Supplementary material for: Comparative analysis reveals distinctive epigenetic features of the human cerebellum
Source: PLoS Genet. 2021 May 6;17(5):e1009506. doi: 10.1371/journal.pgen.1009506 (PMC8101944; doi:10.1371/journal.pgen.1009506)
Supplement: Attachment — Submitted filename: PLoS_Genetics_response_to_reviewer_comments_2021.03.04.docx [file pgen.1009506.s011.docx]

Editor's comments:

The reviewers appreciated the attention to an important topic but identified some concerns that we ask you address in a revised manuscript. We therefore ask you to modify the manuscript according to the review recommendations. Your revisions should address the specific points made by each reviewer.

Reviewer's Responses to Questions

**Comments to the Authors:
Please note here if the review is uploaded as an attachment.**

**Reviewer #1**: The authors have satisfactorily addressed all of my prior comments. In particular, the refocusing of the paper to emphasize the cerebellum very much clarifies and enhances the originality of this study. The additional details provided in the methods are also an excellent improvement. Finally, while the authors do a nice job of highlighting their results and the insights gained from them, they are also clear about the limitations of this work.

We are pleased that the reviewer found our revisions adequate.

**Minor Comments**

[line 472] What does “age class” mean? If this is something different than age in years, please specify this variable in Table S1.

This was an omission. We have added the age class information to Table S1 (and a reference to the table in text).

Some minor typos are present throughout the paper and should be corrected. For example:

• [line 108] “languages areas” should be changed to “language areas”

We have corrected this typo.

• [line 113] remove the comma between “molecular” and “studies”

We meant comparative evolutionary, including molecular, studies (molecular evolutionary studies as a kind of comparative evolutionary study). To clarify better what we meant, we updated the text to: “The DLPFC has long been a focus of comparative evolutionary studies, including molecular analyses.”

• [line 119] add a comma after in-text citation “[27-29]”

We have added this comma.

**Reviewer #3**: The substantially revised manuscript by Guevara et al. presents an partial improvement over the initial submission addressing some points raised by the reviewers, particularly regarding methodological detail. However, some major points were not addressed, including my earlier suggestion to provide a greater focus to this study by identifying the areas of greatest difference between species, narrow down on the most interesting or understudied pathways, and experimentally test whether hypo- or hypermethylation correlates with increased or decreased expression of the genes in question to construct narrow hypothesis around these specific data. While I am very sympathetic to the argument about current limitations placed on most of us, even the existing data without additional experiments may have sufficed to meet part of this suggestion. For instance, I find Table 1 dissatisfying by not listing genes according to relevant gene ontology domains using common tools that may further highlight pathways of relevance.

We misunderstood this comment initially, but appreciate the reviewer’s suggestion for how this could be accomplished with existing data. We have added biological pathway gene ontology terms to Table 1 and have also examined whether humans show unique gene expression patterns in genes annotated by the GO term “chemical synaptic transmission” (GO:0007268), which annotated two of our regDMR-associated genes (new lines 218-223, 564-572).

In addition, the paper’s focus has been shifted by emphasizing the cerebellum as a structure of potential significance to human evolution, but throughout the manuscript and particularly so in the discussion this position is continuously weakened or the evidence supporting a disproportionate significance of the human cerebellum not sufficiently highlighted.

We have revised the manuscript and in particular the discussion to make this argument more consistent throughout (lines 185-187, 201, 231-242, 399-408).

The results section has been shortened to a degree that makes it difficult to follow the process and rationale guiding experiments. Providing some additional detail, currently dispersed in introduction and methods should help. For instance, the results section is initiated by directly presenting the methylation data with no mention of the process leading to these data. While the introduction and methods sections provide more details, some of it has to make it into the results section as well. Specimen selection, amount of tissue collected, lysis procedure, and reference to the chip used for analysis are some essentials to provide context. In the process, please also include a reference to Fig 1 in the results section.

We agree with the reviewer that such information would be useful to readers and have added more details to the Results to provide necessary context (lines 152-164, 182-183, 190-192, 224-226).

Line 165: Many (~7%) of DMRs identified were evident in both brain structures. I don’t think that 7% qualifies as many. I think “some” or a “minority” seems more appropriate.

We have changed “Many” to “some” (new line 173).

Line 169: Overall, correspondence across tissues was high, with average global DLPFC and cerebellum methylation showing a correlation of 0.91-0.92 in all three species.

Line 208: Substantial species divergence in methylation of the cerebellum is notable giving a long standing focus on the prefrontal cortex in comparative neuroscience. Our finding affirms the importance of the cerebellum in human brain evolution.

The above two statements from results and discussion respectively disagree in my opinion. At .91-.92 correlation of both DLPFC and cerebellum between species, both structures show comparable divergence. Also, I am not sure if this level of between species divergence can be considered substantial, as it corresponds well to that seen in blood too.

We agree with the reviewer that these statements seem contradictory. We meant that the PFC is often hypothesized to show greater divergence in humans from other species than other brain structures, but our results in the cerebellum provide evidence to the contrary. We have rephrased the latter statement “Our results showing divergence in methylation of the cerebellum comparable to that in the DLPFC is notable giving a long-standing focus on the prefrontal cortex in comparative neuroscience.” (new lines 231-233).
